# Effectiveness of a chat-bot for the adult population to quit smoking: protocol of a pragmatic clinical trial in primary care (Dejal@)

**DOI:** 10.1186/s12911-019-0972-z

**Published:** 2019-12-03

**Authors:** J. F. Avila-Tomas, E. Olano-Espinosa, C. Minué-Lorenzo, F. J. Martinez-Suberbiola, B. Matilla-Pardo, M. E. Serrano-Serrano, E. Escortell-Mayor, the Group Dej@lo, A. M. Garcia-Mesa, C. Gomez, E. Liebana-Nistal, L. Molina-Alameda, C. Andrade-Rosa, A. Caballero-Jiménez, G. Escribano-Romo, M. Espejo-Romero, A. Ferrero-Brenes, J. L. Hernandez-Garcia, E. Perez-Perez, J. Zambrano-Alvarez, E. Ayuso-Hernandez, M. J. Cuesta-Monedero, F. J. Sanchez-Jimenez, A. Vigil-Escalera, S. Casado-Nistal, M. Garcia-Alva, I. Martin-Gomez, N. Puyo-Rodriguez, A. Rodriguez-Cosio, I. Sanchez-Vazquez, M. A. Alcala-Olmo, M. T. Garcia-Alegre, M. Garcia-Alva, C. Hurtado-Monterrubio, T. Martin-Burillo, C. Moral-Moraleda, E. Ciria Holgado, C. Hermoso-Duran, S. Hernandez-Rodriguez, A. L. Lafraya-Puente, J. L. Melero-Serrano, M. J. Molina-Sanchez, E. Muñiz-Dominguez, C. Saiz-Loma-Osorio, J. Aparicio-Velasco, E. Cerezo-Pascual, L. Fonte-Elizondo, M. I. Gomez-Martinez, C. Marin-Osorio, P. Orozco-Diaz, E. Prieto-Villegas, J. Rodriguez-Donoso, A. Rodriguez-Huete, F. J. Romero-Blanco, J. P. Urbano-Fernandez, S. J. Castillo-Portales, R. Collados-Navas, P. Gutierrez-Sordo, M. B. Martin-Sacristan-Martin, M. Sanchez-Quejido, C. Abad-Schilling, S. Arenas-Gonzalez, M. E. Calonge-Garcia, M. Capitan-Jurado, S. Menendez-Alvarez, M. A. Regalado-Valle, H. C. Alache-Zuñiga, E. Cerrada-Cerrada, R. Garcia-Camps, M. J. Gutierrez-Notario, J. Mendez-Cabezas-Velazquez, J. Molina-Paris, H. Pensado-Freire, G. Viñas-Fernandez, B. Jaenes-Barrios, M. Jurado-Sueiro, M. Ortiz-Sanchez, M. L. Santiago-Hernando, S. Arjona-Perez, I. Delgado-Mellado, R. Diaz-Martin, B. Fandiño-Garcia, M. C. Gomez-Ortiz, M. T. Lopez-Martin-Aragon, C. Martinez-Alvaro, M. A. Miguel-Abanto, M. Noguerol-Alvarez, S. Parra-Roman, Z. Pascual-Garcia, A. L. Ranz-Granados, S. Rey-Rodriguez, C. Sanz-Perez, E. Aguilar-Hurtado, M. Aguilera-Rubio, A. I. Carbonero-Martin, M. Esteban-Garcia, C. Esteban-Peña, M. Fernandez-Sanchez, C. Garcia-Contreras, A. M. Hernandez-Sanchez, M. L. Herrera-Garcia, A. Herrero-Fuentes, M. Oria-Rodriguez, M. S. Parra-Martin, M. J. Rojas-Giraldo, A. Saiz-Ordoño, Y. B. Sanchez-Fernandez, P. Villasevil-Robledo, M. Alonso-Ovies, A. Diaz-Martin, N. Garcia-Arpa, F. Garcia-Rodriguez, O. Gonzalez-Carpio-Paredes, M. J. Grandes-Muñoz, M. Jara-Peñacoba, A. Lopez-Villalbilla, M. J. Parellada-Ruiz-Cuesta, A. I. Rabadan-Velasco, M. Rodriguez-Bastida, C. Sobrado-Cemeño, A. Valdez-Jaquez, S. Vicente-Perez, B. Matin-Porras, A. Gonzalez-Valls, V. Joglar-Alcubilla, J. Santa-Cruz-Hernandez, M. E. Serrano-Serrano, C. Castilla-Alvarez, M. A. Garcia-Abad-Fernandez, M. Garcia-Viada, S. Hernandez-Lachehab, L. Lopez-Kollmer, A. Miguel-Ballano, V. Molina-Barcena, R. Ortega-Pineda, I. Sepulveda-Gomez, P. Cuadrado-Gonzalez, M. J. Jimenez-Moreno, C. Linares-Sanchez, M. A. Pardo-Garcia, F. Sanchez-Martin, R. Simon-Gutierrez, B. Solans-Aisa, T. Francisco-Romanillos, A. Garcia-Ortega, P. Hernandez-Garcia-Alcala, F. Huguet-Vivas, M. L. Juarez-Zapatero, C. Lopez-Garcia, M. J. Manzano-Martin, J. A. Martinez-Torres, M. Miguel-Garzon, M. P. Rodrigo-Rodrigo, M. A. Ruano-Dominguez, A. Serna-Urnicia, N. Altares-Arriola, R. Aranzo-Pacheco, C. Casado-Rodriguez, C. Cascao-Moutinho-Pereira, M. Escudero-Araus, C. Fuentes-Manrique, S. Guijarro-Abanades, P. Hombrados-Gonzalo, A. M. Lopez-Carabaño, M. L. Meiriño-Perez, G. D. Moneva-Vicente, I. Noguera-Martinez, M. Perez-Fernandez, M. Robres-Olite, T. Rubio-Rubio, M. F. Venegas-Gato, F. Bernal-Hertfelder, M. Blas-Escribano, M. M. Castro-Fouz, B. Castro-Sanchez, L. Dominguez-Perez, P. A. Garcia-Gomez, A. Herrero-Dios, M. Lor-Leandro, I. Lozano-Martin, S. Pacho-Pinto, R. Pastor-Sanchez, M. D. Moreno-Chaparro, E. Yagüe-Fernandez, M. J. Lopez, A. Mateo-Madruga, J. L. Palancar-Torre, P. Pumar-Sainz, C. Aragon-Marente, M. A. Gomez-Medina, J. A. Granados-Garrido, I. Gutierrez-Sanchez, M. J. Heras-Alonso, M. J. Lopez-Gomez, B. Martin-Peces, F. J. Martinez-Suberbiola, A. Medran-Gomez, G. Nuevo-Rodriguez, S. Ochoa-Vilor, C. Vargas-Machuca, O. Fernandez-Montes-Lopez-Morato, R. Jimenez-Rojas, C. Marcos-Frutos, C. Minguet-Arenas, C. Minue-Lorenzo, V. Ruiz-Pascual, I. Sanchez-Fonseca, J. M. Sanchez-Gonzalez, F. Alba-Gomez, J. F. Avila-Tomas, A. Cayuela-Mate, E. Cidoncha-Calderon, A. B. Cifuentes-Muñoz, R. M. Fernandez-Garcia, J. L. Gala-Paniagua, A. Gongora-Marin, P. Herrero-Municio, E. Moreno-Chocano-Garcia-Carpintero, S. Perez-Cuadrado, A. Redondo-Horcajo, C. Vicente-Sanchez, R. M. Villena-Romero, O. Hakami-Hakami, M. M. Alvarez-Villalba, M. J. Bedoya-Frutos, J. Camarero-Palacios, M. Escobar-Gallegos, A. Hormigos-Agraz, J. Innerarity-Martínez, M. T. Lopez-Lopez, B. Perez-Ballesto, E. Perez-Gutierrez, M. P. Tardaguila-Lobato, R. Terron-Barbosa, A. Ballarin-Gonzalez, Y. Fernandez-Fernandez, I. Ferrer-Zapata, E. Gomez-Suarez, M. Lamonaca-Guasch, F. Morales-Ortiz, C. Pelaez-Laguno, J. L. Quintana-Gomez, E. Rioja-Delgado, J. I. Aza-Pascual, D. Fuente-Arriaran, R. A. Garcia-Perez, A. Garriz-Aguirre, S. Gomez-Diaz, P. Gonzalez-Palacios, N. Gonzalez-Sanchez, G. Peralta-Alvarez, C. Sanz-Velasco, J. Vazquez-Gallego, C. Vazquez-Garcia, C. Fernandez-Duran, N. Peña-Anton, J. M. Blanco-Canseco, I. Gamez-Cabero, E. Minguela-Puras, J. Serrano-Vega

**Affiliations:** 1 Universidad Rey Juan Carlos, Madrid, Spain; 2 Healthcare center Sta. Isabel, Madrid Regional Health Service, 28911, Leganés, Madrid, Spain; 3 Healthcare center Los Castillos, DAO, Madrid Regional Health Service, Alcorcón, Madrid, Spain; 4 Healthcare center Perales del Río, DAC, Madrid Regional Health Service, Getafe, Madrid, Spain; 5 Healthcare center Guayaba, DAC, Madrid Regional Health Service, Madrid, Spain; 6 Healthcare center Panaderas, DAO, Madrid Regional Health Service, Fuenlabrada, Madrid, Spain; 7 Healthcare center Los Fresnos, DAE, Madrid Regional Health Service, Torrejón de Ardoz, Madrid, Spain; 8 Gerencia Asistencial de Atención Primaria (GAAP), Madrid, Spain; 9 Health Services Research on Chronic Patients Network (REDISSEC), Madrid, Spain

**Keywords:** Smoking, Tobacco cessation, Primary care, Cell phone use, Chat-bot, Dialog systems

## Abstract

**Background:**

The wide scale and severity of consequences of tobacco use, benefits derived from cessation, low rates of intervention by healthcare professionals, and new opportunities stemming from novel communications technologies are the main factors motivating this project. Thus, the purpose of this study is to assess the effectiveness of an intervention that helps people cease smoking and increase their nicotine abstinence rates in the long term via a chat-bot, compared to usual practice, utilizing a chemical validation at 6 months.

**Methods:**

Design: Randomized, controlled, multicentric, pragmatic clinical trial, with a 6-month follow-up. Setting: Healthcare centers in the public healthcare system of the Community of Madrid (Madrid Regional Health Service). Participants: Smokers > 18 years of age who attend a healthcare center and accept help to quit smoking in the following month. *N* = 460 smokers (230 per arm) who will be recruited prior to randomization. Intervention group: use of a chat-bot with evidence-based contents to help quit smoking. Control group: Usual treatment (according to the protocol for tobacco cessation by the Madrid Regional Health Service Main variable: Continuous nicotine withdrawal with chemical validation (carbon monoxide in exhaled air). Intention-to-treat analysis. Difference between groups in continuous abstinence rates at 6 months with their corresponding 95% confidence interval. A logistic regression model will be built to adjust for confounding factors. Results: First expected results in January 2020.

**Discussion:**

Providing science-based evidence on the effectiveness of clinical interventions via information technologies, without the physical presence of a professional, is essential. In addition to being more efficient, the characteristics of these interventions can improve effectiveness, accessibility, and adherence to treatment. From an ethics perspective, this new type of intervention must be backed by scientific evidence to circumvent pressures from the market or particular interests, improve patient safety, and follow the standards of correct practices for clinical interventions.

**Trial registration:**

ClinicalTrials.gov, reference number NCT 03445507.

## Background

Tobacco use is the main cause of preventable morbidity and mortality in the world, directly accounting for five million deaths yearly [1]. The number of smokers who attempt quitting on their own every year is high, but only 2–3% remain abstinent after 12 months [2]. Healthcare professionals are very effective and efficient in their interventions on the smoker, with over threefold success rates of long-term abstinence. Combining behavioral and pharmacological treatment yields the best results [2–4].

However, only 1 in 20 attempt to quit is supervised by a professional [2]. In the public healthcare system of the Community of Madrid (Madrid Regional Health Service), 84% of smokers who attended an outpatient consultation in 2008 did not receive advice for tobacco cessation [5], a figure similar to those observed in other countries [6, 7]. Factors related to these low intervention rates have been identified, and these include lack of training for healthcare professionals and their perception that such interventions are not useful and they lack the time to implement them [8].

A possible solution is developing effective, brief, and simpler interventions, such as the “Very Brief Advice “that consists of offering the patient help to quit smoking regardless of their motivation. Many patients respond positively to this proposal, even those who had not considered making an attempt previously to the offer [9]. Compared to the usual intervention, this results in over 50% more attempts to cease smoking [10]. However, to achieve this, a resource backed by scientific evidence and easy to access must be available, such as the tobacco cessation consultations in the British National Health System (NHS).

The boom in information and communication technologies, like the internet or smartphones, open up new therapeutic perspectives. In 2016, there were 7 billion customers of mobile phone services, almost 97% of the population worldwide. Mobile broadband has continuously grown, with 84% of the world population using it [11], and the smartphone has become the main and most accessible personal computer in the majority of countries. On the other hand, patients aspire to play a greater role in their health management and increasingly search for more information in the internet (Table 1). The resulting opportunities create a new framework to empower the patient and improve clinical outcomes and health expenditure [12].

**Table 1:** Characteristics of the web search for health information

Advantages	Downsides
Ubiquity	Unequal quality of information
Immediacy	An identified source cannot always be found
Ability to access specific population segments	The date when the content was updated cannot always be found
Automation of messages	Need to adapt the message to the targeted population
Possibility of contact and learning among peers	Risk of information overload for the patient

Translation from: Casado, S. El Papel de la Información en el empoderamiento del paciente en Basagoiti I. Alfabetización en salud. De la información a la acción [pdf]. Valencia: ITACA/TSB; 2012. ISBN: 978–84- 695-5267-4 Available at http://www.salupedia.org/alfabetizacion/

In terms of scientific evidence, a systematic review of 59 clinical trials on interventions to quit smoking via smartphones [13] concluded that text messages double the success rates of nicotine abstinence with biochemical validation, showing a risk ratio (RR) of 2.16 (Confidence interval -CI- 95%: 1.77–2.62). The relevant Cochrane review [14] comprised 12 clinical trials that included 11,885 patients, with a combined RR = 1.7 (CI 95%: 1.46–1.90) for abstinence at 6 months, and a RR = 1.83 (CI 95%: 1.54–2.19) using the data of the six clinical trials that chemically validated the abstinence, both compared to usual practice. The evaluated trials were based on interventions mainly using Short Message Service (SMS), although one employed videos, and all were conducted in high-income countries with strong policies for tobacco control. They also did not find clinical trials, published or in-progress, studying the effectiveness of applications (app) for mobile phones or tablets, despite their proliferation.

In a 2013 review about the adequacy of scientific evidence for existing apps to aid people cease smoking in the USA at the time, Abroms et al. [15] could not find any that followed science-based recommendations by clinical practice guidelines and warned about the potential negative effects on the population’s health.

In 2017, a new review [16] aimed to assess the scientific content of the most used commercial apps in the USA for helping to quit smoking as well as the ones available. Only six apps were identified as being partially science-based, of which three (50%) were available in at least one app store, and just two of the top 50 recommended apps in app stores (4%) had some scientific basis, but it was not possible to differentiate them from those not based on scientific evidence.

A chat-bot is not a software that needs to be installed in a smartphone or tablet, it respects the privacy of the patient scrupulously, and its learning curve is very short [17]. This computer software has a conversation interface that can both answer questions posed by the user in a natural language (that commonly used by people with all their variants) and ask them questions [18]. Although it is not a new technology, the recent technical revolution for the interpretation of natural languages, together with the above-mentioned advantages and certain downsides of apps, make chat-bots ideal tools for the purposes of this intervention. Since chat-bots “understand” requests expressed with the complexity and variability of human language, they provide a component of technological “humanization” that other interfaces based on menus and chat-buttons lack. This way, they are capable of returning a personalized answer and add a component of user loyalty and usability of the tool [18, 19].

The chat-bot to be evaluated in this trial has been specifically designed by experts in tobacco addiction and artificial intelligence incorporating gamification, cognitive-behavioral, motivational, problem-solving, and relapse-preventing components. Such components form an integral part of science-based interventions recommended by clinical practice guidelines [3, 4].

We aim to assess whether the effectiveness of the usual intervention in primary care can be improved [2] by providing healthcare professionals with guidance to help patients accept the offer to quit smoking [10], so that their motivation to intervene on their patients will increase by decreasing their workload. We also estimate that this type of intervention will improve the accessibility of patients to an evidence-based treatment.

The wide scale and severity of consequences of tobacco use, benefits derived from cessation, low rates of intervention by healthcare professionals, and new opportunities stemming from novel communications technologies in addition to the absence of scientific evidence on its effectiveness and the lack of such tools designed by experts according to the guidelines of the evidence-based clinical practice guidelines are the main factors motivating this project.

## Methods

### Aims

#### Main objective

To assess the effectiveness of an intervention in primary healthcare to help smokers cease their tobacco addiction via a chat-bot for smartphones compared to usual practice, as measured through biochemical validation of continuous abstinence at 6 months.

#### Secondary objectives

1. To assess the intervention effectiveness compared to usual practice as measured through self-reported continuous abstinence at 6 months.
2. To assess the intervention effectiveness compared to usual practice in terms of the improvement in their quality of life measured by the EuroQol-5D-5 L questionnaire.
3. To evaluate the cost-utility ratio of the intervention compared to usual practice in primary healthcare.
4. To assess the intervention effectiveness in terms of improved adherence to pharmacological treatment for tobacco cessation.

#### Design

**Fig. 1:**
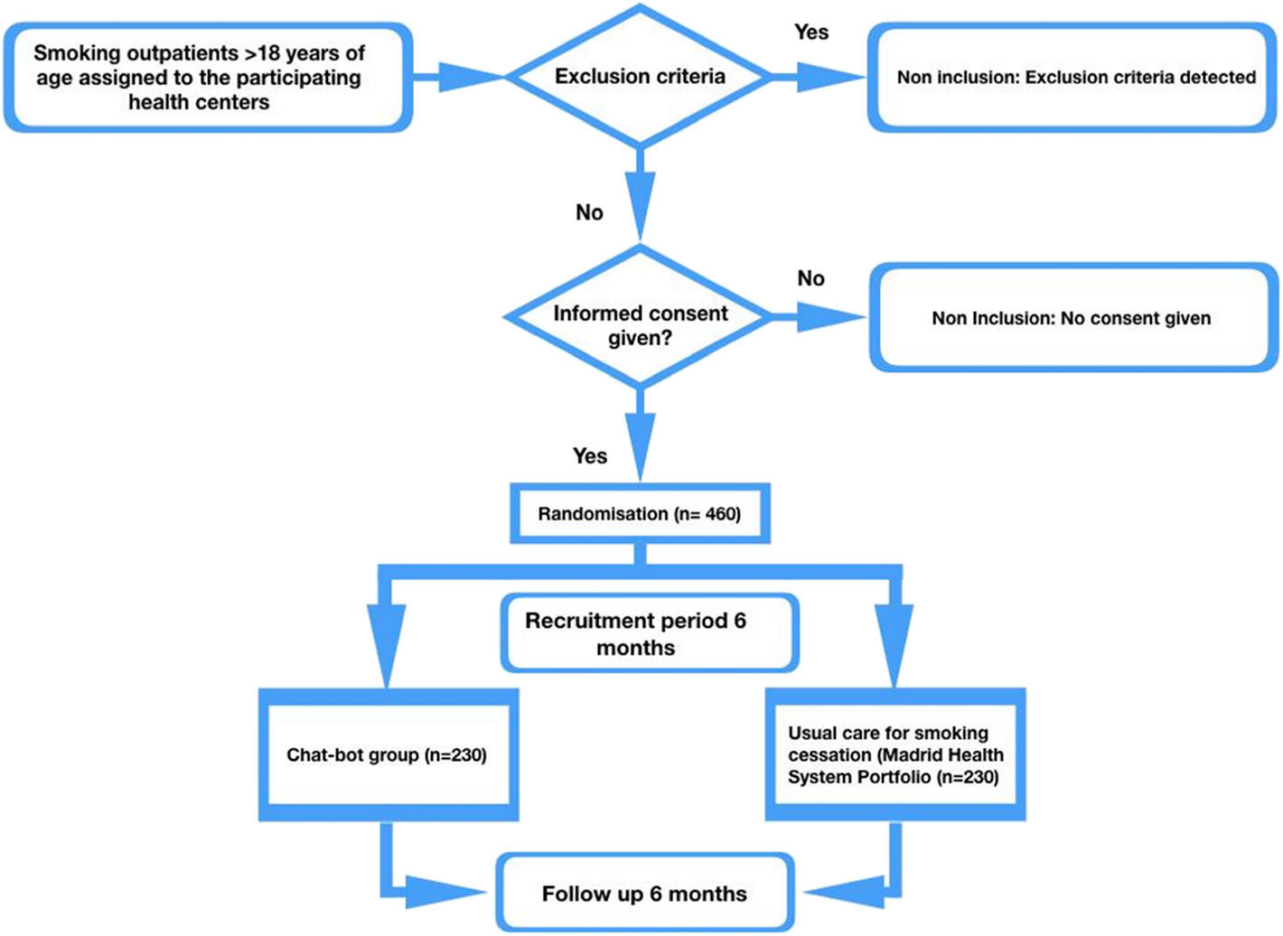
Protocol

**Fig. 2:**
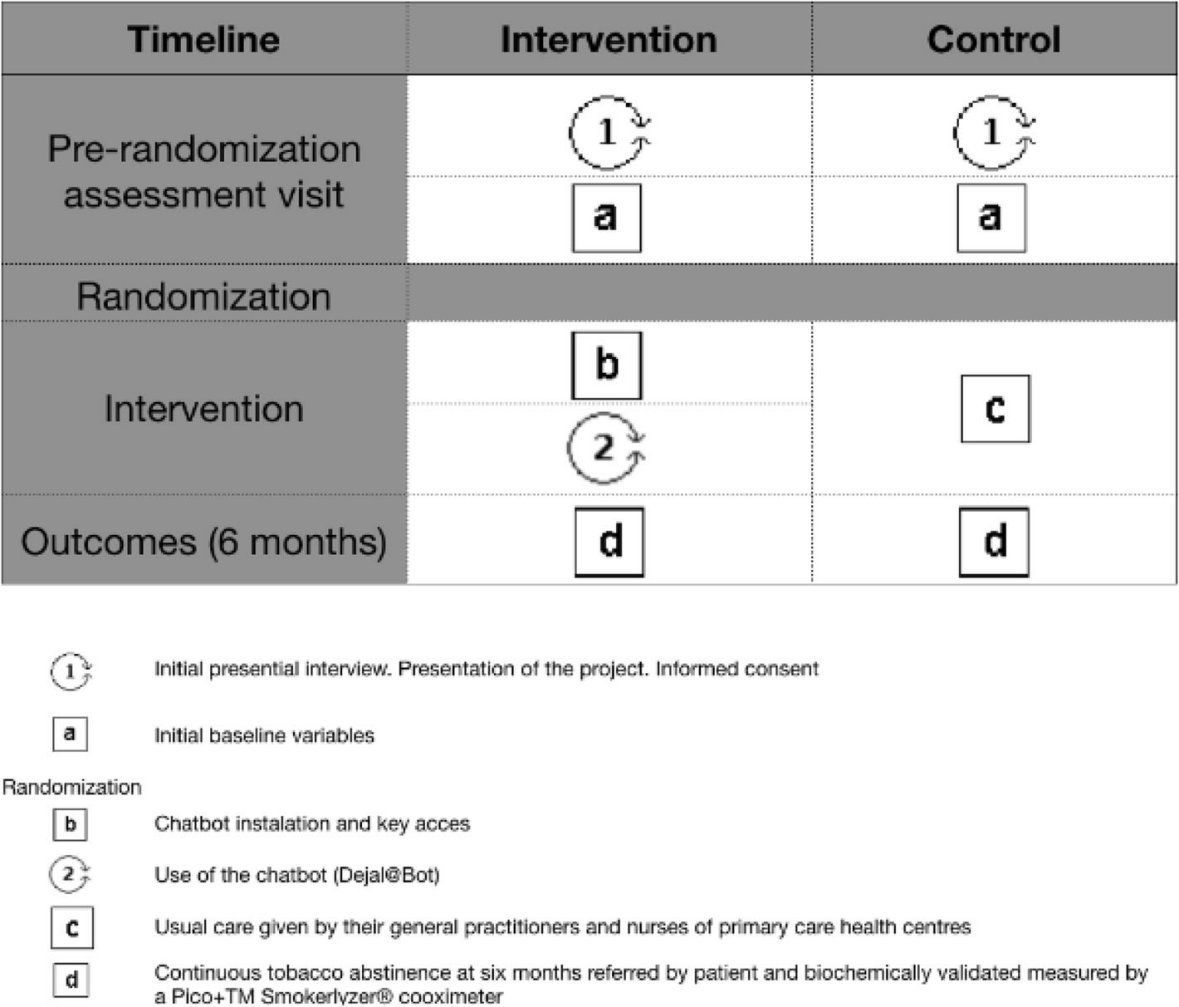
Pat plot intervention Dej@lo

The clinical trial will be pragmatic, randomized, controlled, and multicentric. It will last one year, six months for recruiting and six months of follow-up. A cost-utility study will be conducted from the perspective of the funder for a 1-year timeframe. (Figs. 1,2)

#### Setting

Primary healthcare centers in the Madrid Regional Health Service

#### Participants

Patients > 18 years of age who smoke and attend their primary care doctor or nurse consultation during the inclusion period.

### Criteria for inclusion

1. Healthcare centers: Doctors and nurses from 34 healthcare centers in the Madrid Regional Health Service who agreed to participate, amounting to a total of 248 participants.
2. Patients: Being > 18 years of age; having smoked more than one cigarette per day during the last month; accepting the offered help to quit smoking in the following month; owning a smartphone where a messaging app can be installed; being reachable during the six months following the beginning of the intervention; agreeing to participate and signing informed written consent.

The criteria for exclusion are: Significant communication barriers; Addiction to other substances; And participating in another dishabituation program or clinical trial during the study period.

#### Sample size

The sample size was calculated based on the outcome of a clinical trial recently conducted of usual clinical care in Madrid public health system that employs a similar definition of continuous abstinence as our study [5]. It detected an abstinence rate of 9.6% at 6 months in the control group. We estimate that the intervention to be assessed will double this fig [13, 14]..

Based on these data, and considering a type I error of 5% and a power of 80%, the required number of patients will be 418. After estimating 10% of losses to follow-up, the required sample size is 460 smokers (230 in each group).

#### Recruitment

Each healthcare professional will select patients who smoke, attend the health center for any reason within the recruitment period, and meet the inclusion criteria. They will inform patients about the characteristics of the trial and offer them to participate. Those who accept will be asked to provide informed written consent. In case they decline, their gender, age, and reason for declining will be recorded.

Various strategies will be implemented in order to increase adherence to the protocol by participating researchers, such us personal follow-up upon achievement of protocol objectives and acknowledgement of their effort via e-mail, and offers of certified training sessions.

Randomized allocation: Subjects will be allocated into the intervention or control group by simple randomization using a software installed in the data collection notebook (DCN). This will be done following the inclusion of patients and data collection in the DCN at the initial visit, which guarantees the masking of the randomization sequence to the professionals recruiting patients. The group assigned to the chat-bot will be the intervention group and those treated with usual care by their doctor or nurse will be the control group.

#### Criteria for abandonment and withdrawal

Participants will be able to abandon the study at any time and researchers will be able to withdraw any patient under the following circumstances: not meeting the inclusion criteria; Onset of a severe illness during the clinical trial; Or adverse event, and inability to comply with the study requirements.

The number of abandonments and withdrawals, as well as the reasons for them, will be recorded. Withdrawn patients will not be excluded from the intention-to-treat analysis.

#### Masking

The design of the study does not allow for masking the patient to the received treatment. However, this limitation is compensated by the objective measure of the main outcome variable (smoking abstinence) and the random allocation of patients into study arms. Additionally, researchers in charge of the statistical analysis will be blinded to the identity of patients in each treatment group.

## Interventions

The intervention strategy to follow in both arms is based on the five A’s of the clinical practice guideline by the Agency for Healthcare Research and Quality [4]: ask, advise, assess, assist, and arrange. This way, all patients will be questioned in person about their tobacco addiction, receive advice from their doctor or nurse to cease smoking, and their willingness to quit will be evaluated. Patients who accept the offered help for the month following consultation will be randomly assigned to the intervention or control group, where they will receive a personal intervention combining behavioral and pharmacological treatment based on scientific evidence [2–4]. The intervention will be organized into several follow-up sessions, either on-line via the chat-bot (in which case the patient can access the intervention at their convenience) or face-to-face with their healthcare professional in the healthcare center.

### Intervention group

Patients assigned to the intervention group will use a chat-bot whose script for the smoking cessation process has been developed using contents based on current evidence. Subjects will download a messaging application on their smartphone for this purpose and will access the chat-bot with an assigned personal password. The chat-bot will guide them through all the stages of the dishabituation process. The chat is bidirectional, employs multiple media formats, and provides patients with automatic, science-based advice on cognitive-behavioral, motivational, relapse-preventative, and problem-solving techniques. This will take place with different periodicity depending on the quit date and other characteristics of the patient (personal choice, type of tobacco use, personal risk situation, prescribed medication, abstinence-related symptoms, and the evolution of coping with abstinence). The chat-bot also offers information about useful medication to quit smoking prescribed by their healthcare professional, and will recommend how to face problems related to the cessation process through advice and relaxation exercises available in different formats, such as video, computer graphics, games, and web links. Additionally, the chat-bot incorporates gamification elements (games for adults for acquiring new knowledge and skills), including a system to earn scores and badges that will provide access to specific information depending on the abstinence period and the needs of the individual. Specific aspects of the game mechanics (score, levels for different abstinence periods) and dynamics (rewards, acknowledgment) will be developed. Contact with the patient will begin at day ^− 15^, where the above-mentioned strategies will be established, and daily interactive appointments will be set until the quit date. From this point, subjects will receive encouragement and acknowledgement messages that will be spaced out gradually until six months of abstinence. The patient can contact the chat-bot at any time, so the number and total time of interactions are a priori unknown and will be recorded as secondary variables. In terms of proactive contacts, the patient can choose their timing and frequency.

#### Control group

Patients assigned to the control group will receive the usual practice for smoking cessation, namely the different interventions by primary care nurses and physicians included in the services portfolio of the Madrid Regional Health Service (Service 415, Care for adult smokers) that consist of one or two visits prior to the quit date, follow-up visits after one week and one month, and subsequent control visits depending on whether the opportunity arises, the professional’s criteria, and the patient’s needs.

#### Variables

The collaborating healthcare professionals will deliver their data prior to the beginning of the study. The recruiting participants will record patient data and will be also responsible for their follow-up. All the information will be recorded on a form created for the trial (DCN). Each collaborator will access the form from their personal computer via the project’s website, using a code for personal identification. Two visits have been defined for data collection of patients: basal and at 6 months.

#### Main outcome variable

Continuous tobacco abstinence at 6 months (yes/no). In accordance to the recommendations by “the Russell Standard” [20], abstinence is confirmed when the patient declares to have smoked five or less cigarettes since the beginning of the abstinence period and after being validated with a co-oxymeter Pico+TM Smokerlyzer® that reads < 10 carbon monoxide particles per million (ppm) in exhaled air.

#### Secondary outcome variables

Quality of life of the patient measured via the EuroQol-5D-5 L, a generic questionnaire in their Spanish version validated for our setting [21] that comprises two parts. The first part consists of five questions on the health condition of subjects to explore five dimensions: mobility, self-care, usual activities, pain/discomfort, and anxiety/depression. Each dimension is measured on a 5-level scale. From these five questions, a single weighed score is obtained, the so-called utility index. The higher the score the better the health condition. To obtain this index, the algorithm proposed for Spain has been employed [22]. The second part consists of a visual analog scale (VAS) ranging from 0 (worst possible condition) to 100 (best possible health condition). The intergroup differences in the utility score at 6 months will be used to construct the quality-adjusted life years (QALYs) gained with the intervention.

#### Employed time

Number of consultations and total time (in minutes) will be recorded in the DCN by the healthcare professional and automatically registered from the chat-bot.

#### Cost-utility

Costs derived from the intervention will be calculated via a proxy of their cost-opportunity. The price of avoided visits, calculated from the average salary of the health worker’s professional category, will be categorized as “saved costs”. An uncertainty threshold will be estimated for all the applied costs comprising the time range attributable to each activity. The calculation of benefits will be done based on the QALYs, quantifying the total gain from the EuroQol-5D-5 L utilities in the intervention group at 6 months and assuming they will stay constant for one year.

#### Adherence to treatment

Number of weeks the patient gets pharmacological treatment prescribed by their doctor or nurse, as recorded by the professional in the DCN during each visit or automatically registered by the chat-bot.

#### Explanative and adjusting variables

Patient-related socio-demographic variables (first level): 1) age (years); 2) gender (M/W); 3) socio-economic status (monthly income expressed as a multiple of the minimum wage); 4) educational level (no education/primary education/secondary education/university education); 5) nationality; 6) number of cigarettes/day; 7) number of previous attempts to cease smoking; 8) basal co-oximetry in exhaled air (ppm); 9) quit date; 10) level of nicotine dependence (brief form of Fageström Test); 11) continuous cannabis use (defined as at least once per week for the last month).

Healthcare centers and healthcare professionals (second level): 1) gender (M/W); 2) age (years); 3) professional category (medicine, nursing); 4) graduation year; 5) average workload (measured as the average daily consultations per health worker during the year prior to the trial).

### Statistical analysis

The results will be presented in accordance with the guidelines by the CONSORT declaration [23].

#### Description of basal characteristic

A descriptive analysis of the demographic and basal characteristics of subjects in both groups will be conducted: quantitative variables will be described by their measures of central tendency (mean or median in the case of asymmetric distributions) and their dispersion (standard deviation or interquartile range for normal or non-normal distributions, respectively). Qualitative variables will be described by their proportions with their confidence intervals set at 95% (CI 95%).

#### Basal comparison

Intergroup differences will be analyzed using statistical tests for independent samples (Student’s t-test or chi-squared test), whereas a test for related samples (repeated measures ANOVA) will be employed for differences within each group and across visits.

#### Main effectiveness analysis

Intergroup differences in the continuous abstinence rates at 6 months (main variable) will be analyzed with their CI 95%. Bivariate analysis: a Student’s t-test, or the Mann-Whitney U-test in case the hypothesis of data normality is rejected, will be used to compare the outcome between groups in the case of quantitative variables. For qualitative variables, either Pearson’s chi-squared test or Fisher’s exact test, when applicable, will be used. After adjusting for possible confounding factors, the odds ratio of the association will be calculated using a logistic regression model where the dependent variable will be the continuous abstinence (yes/no) and the independent variables will be patient-related variables and the study group. In all cases, statistical significance will be set at *p* < 0.05. Gained QALYs will be estimated at a population level with their relevant CI 95% via parametric analysis methods and bootstrap techniques. Cost-utility ratio: this aim is exploratory (there is no specific design for it). The cost-utility ratio will be calculated dividing the overall cost by the sum of potential gains expressed in QALYs. For normally distributed data, a multivariate sensitivity analysis will be conducted, making the costs fluctuate within the constructed range of uncertainty. For the same distribution, we will also make the benefits (QALYs) fluctuate within the CI 95%. In all cases, the analysis will be for intention-to-treat. The number of withdrawals, losses to follow-up, and subjects not complying with the protocol will be recorded together with the relevant reasons. Losses to follow-up will be considered as smokers in the analysis [24]. A participant will be considered lost to follow-up after missing two appointments, failing to be contacted by phone in at least two occasions, and having no registered explanation for it. Missing information on professionals and/or patients will be replaced using values from the baseline data.

## Discussion

Both the healthcare professionals accepting to participate in a clinical trial and the recruited patients may be more interested in the subject of the trial, sharing an increased awareness and motivation for the studied health issues, which can lead to a bias. Although this can result in better outcomes compared to trials with less selective recruiting processes, it would have a conservative effect (namely, it would decrease the magnitude of the intergroup difference).

Additionally, participating collaborators may modify or improve their prescribing habits due to their awareness of being under observation (known as the Hawthorne effect). This may reduce differences between the intervention and control groups in terms of suitability of prescription.

A design by clusters was not chosen in this intervention, since the possible influence among smokers in the same healthcare center would be residual given that the app is personal and with a restricted access for the patient. This circumvents other biases resulting from variability in the clinical practice of included professionals and their basal knowledge on the subject of the intervention.

The intervention cannot be masked, so a blinded evaluation of the main outcome variable has been designed. The researchers in charge of the data analysis and interpretation will be unaware of the allocation arm of patients.

Although the assessment of the effect of the intervention on quality of life is not confirmatory and the expected changes are limited given the short period of time of the trial, we consider it essential to include this outcome variable in the analysis since intergroup differences can be detected even if they are not strongly significant. Additionally, measuring changes in quality of life allows for incorporating outcome variables that are self-reported by the patients, which facilitates the calculation of utilities in the cost-utility analysis. This will enable to overcome some deficiencies and limitations observed in previous interventions by Patterson et al. [24].

The chat-bot can potentially increase the effectiveness of the usual intervention for several reasons: on the one hand, it will provide primary care professionals with a very brief advice that will direct them to offer help for cessation [10], which has proven its high effectiveness for generating attempts to quit; on the other hand, it will at the same time reduce their workload, increasing their motivation to intervene on their patients. Additionally, the overall time employed in the dishabituation process would significantly increase, a variable that has been identified as one of the main predictors for continuous abstinence [2]; lastly, reminding and monitoring the use of pharmacological treatment will predictably improve adherence to it and, therefore, abstinence rates.

We estimate that this intervention will also show improved accessibility, since it enables people to access an effective program for quitting smoking even when physical limitations, schedule conflicts, or proximity issues exist that hinder face-to-face consultations.

On the other hand, our pragmatic design [25] will allow testing the usage of the chat-bot in conditions that closely mimic real life. Both the recruitment of patients and training of healthcare professionals, as well as the information given to patients and characteristics of healthcare centers, were the same in both arms. Other aspects denoting its pragmatic design were the criteria for inclusion, flexibility in applying the intervention, comparison with usual practice, absence of formal visits set by protocol, and intention-to-treat analysis.

It is essential to provide evidence on interventions directed to outpatients who smoke and that, owing to new information technologies, require less intervention by healthcare professionals. Given the spread of smartphones worldwide, there has been a great boom of apps that facilitate information on health issues and disease control and treatment. However, new approaches based on information and communications technologies appear at a much higher speed than controlled, high-quality trials.

The prediction is that this trend will increase due to the expanding number of users and the redundancy of different approaches to manage one’s healthcare on account of the new available functions of technological advances, so it is important to study why this is happening and remedy it in order to prevent ethical dilemmas and harm to patients. In order to help identify effective interventions, we strongly believe that clinical trials must be performed to evaluate new technologies and their related approaches to help smokers quit, just as they are performed for pharmacological drugs.

## Data Availability

The datasets used and/or analyzed during the current study are available from the corresponding author on reasonable request.

## Abbreviations

App: Mobile Application
CI: Confidence interval
CO: Carbon Monoxide
DCN: Data Collection Notebook
ERDF: European Regional Development Fund
FIIBAP: Fundación para la Investigación e Innovación Biomédica en Atención Primaria
FIS: Fondo de Investigaciones Sanitarias
NHS: British National Health System
PIN: Personal Identification Number
PPM: Particles Per Million (in exhaled air)
QALYs: Quality-Adjusted Life Years
RR: Risk ratio
SMS: Short Message Service. Text messages
VAS: Visual Analog Scale
